# Haustorium Formation and Specialized Metabolites Biosynthesis Using Co-Culture of *Castilleja tenuiflora* Benth. and *Baccharis conferta* Kunth

**DOI:** 10.3390/biology14080990

**Published:** 2025-08-04

**Authors:** Annel Lizeth Leyva-Peralta, José Luis Trejo-Espino, Guadalupe Salcedo-Morales, Daniel Tapia-Maruri, Virginia Medina-Pérez, Alma Rosa López-Laredo, Gabriela Trejo-Tapia

**Affiliations:** 1 Departamento de Biotecnología, Centro de Desarrollo de Productos Bióticos, Instituto Politécnico Nacional, Yautepec 62739, Morelos, Mexico; annie.leyva.peralta@gmail.com (A.L.L.-P.); jtrejo@ipn.mx (J.L.T.-E.); gsalcedo@ipn.mx (G.S.-M.); dmaruri@ipn.mx (D.T.-M.); vmedinap@ipn.mx (V.M.-P.); arlopez@ipn.mx (A.R.L.-L.); 2 Red de Biotecnología, Red de Medio Ambiente, Instituto Politécnico Nacional, Yautepec 62739, Morelos, Mexico

**Keywords:** caffeic acid, caffeoylquinic acids, flavones, hemiparasitic plant, plant–plant interaction, verbascoside

## Abstract

In this study, an in vitro co-culture system of *Castilleja tenuiflora* and its host, *Baccharis conferta*, was used, and the impact of their interaction on specialized metabolite content was analyzed. After 4 weeks of co-culture, haustoria formation was verified through environmental scanning electron and confocal microscopy, confirming the successful establishment of the plant–plant interaction. Shoot height and biomass of the aerial part of the hemiparasite were not affected significantly by co-culture. However, root biomass increased by 53% compared to individually grown plants. Co-culture significantly reduced the host’s root length without negatively affecting its overall growth or survival. Phytochemical profile alterations were observed in both species. For *C. tenuiflora*, the lignans sesamin and eudesmin are proposed as differentially accumulated metabolites, while in *B. conferta*, the caffeoylquinic acid, 4,5-di-*O*-caffeoylquinic acid, and the flavonoid acacetin were expressed differently. The development and chemical profiles of *B. conferta* and *C. tenuiflora* change when they grow in a co-culture because of the host–parasite interaction. Here, we report the feasibility of using a hemiparasite–host system to investigate more profound research questions. Future biotechnological applications of this system include elucidating the genetic regulators involved in haustorium formation, as well as optimizing environmental and physiological conditions to enhance its biosynthetic capacity for the production of specialized metabolites with therapeutic value.

## Simple Summary

In the natural environment, organisms exhibit intricate interconnections in various forms; one notable interaction among plant species is hemiparasitism. A range of physiological processes and morphological alterations occurs within the species engaged in this relationship. To investigate certain facets of this phenomenon, an in vitro co-culture of the hemiparasitic plant *Castilleja tenuiflora* and its host species, *Baccharis conferta*, was employed, resulting in the successful induction of haustoria. This study examined haustoria formation, alterations in root biomass, and the production of chemically significant compounds. In vitro co-cultivation of *C. tenuiflora* and *B. conferta* highlights the significance of developing systems that enhance our comprehension of plant–plant interactions.

## 1. Introduction

Parasitism is a highly specialized survival strategy in plants that enables them to obtain resources from their hosts. Parasitic plants are classified into holoparasites and hemiparasites based on their nutrient dependency on hosts. Hemiparasites can photosynthesize but acquire inorganic nutrients from their hosts to gain a competitive advantage [1]. This interaction necessitates physical attachment between the host and the parasite, either aboveground (shoot-to-shoot) or belowground (root-to-root), through a unique organ known as the haustorium. This multifunctional organ facilitates attachment, penetration, and connection with the host, creating a continuum for exchanging water, nutrients, hormones, proteins, and nucleotides [2]. Haustoria formation is a highly regulated process triggered by recognizing host-derived chemical signals known as haustorium-inducing factors (HIFs). Cytokinins, phenolics, quinones, and ROS have been identified as HIFs [2,3].

Although parasitic plants that negatively impact agriculture, such as *Striga* spp. and *Phelipanche* spp., have been extensively studied to develop management and control strategies, the hemiparasitic interactions among species with potential therapeutic uses, like medicinal plants, have received limited attention [4,5].

*Castilleja tenuiflora* Benth. (Orobanchaceae) is a generalist facultative root hemiparasite with medicinal uses [6]. Research on its chemical composition has shown that it accumulates terpenes, including iridoid glycosides, phenylethanoid glycosides (PhGs), flavonoids, and lignans [7,8]. Pharmacological studies have confirmed its efficacy as an antioxidant, anti-inflammatory, cytotoxic, gastroprotective, and antidepressant agent [8,9]. Given its therapeutic potential, we are interested in developing biotechnological strategies to ensure a sufficient supply of plant material as a source of specialized metabolites. However, its hemiparasitic nature imposes certain limitations. In a previous study, we described *C. tenuiflora* as a facultative parasite of *Baccharis conferta* Kunth (Asteraceae), detailing the anatomy of the haustorium and how carbon levels in both plants are affected by their parasitic interaction [10]. To induce *C. tenuiflora* haustoria under laboratory conditions, we applied HIFs to in vitro plantlets, including H_2_O_2_ and phenolics [11]. Developing a hemiparasite–host system is essential to deepen our understanding of host–parasite interactions.

*Baccharis conferta* is a shrub that serves as a nurse plant for other species growing beneath its canopy [12]. It has medicinal properties, and its phytochemistry and pharmacology have been investigated. It synthesizes flavonoids such as cirsimaritin and terpenes like bacchofertin, exhibiting anti-inflammatory and anti-arthritic activities [13]. In previous studies, we reported the establishment of protocols for in vitro propagation, including co-culture with *C. tenuiflora*, along with the production of caffeoylquinic acids in callus cultures [14,15].

Research has primarily focused on applying HIFs to the parasitic plant to initiate, characterize, and understand haustorium formation [3,4]. Studies on the effects of plant–plant interactions in a (hemi) parasite–host system on physiology and specialized metabolism are limited. For instance, the chemical profile of *Helicanthes elasticus* was influenced by the hosts on which it grew [5]. *Cuscuta campestris* induced metabolic changes, such as a reduction in sesquiterpene and monoterpenoid levels in its host *Artemisia campestris* [16]. This study investigated plant–plant interactions on specialized metabolite content using a co-culture system of *B. conferta* and *C. tenuiflora*. The study findings will enhance the understanding of hemiparasites with biotechnological and therapeutic significance.

## 2. Materials and Methods

### 2.1. Plant Material

*Castilleja tenuiflora* plants were grown from shoots that were twenty-one days old (3 cm) in 50 cm test tubes containing 10 mL of Schenk and Hilderbrandt (SH) culture medium, supplemented with 30 g L^−1^ of sucrose, 2.2 g L^−1^ of phytagel^®^ (Sigma-Aldrich, St. Louis, MO, USA), and 10 µM of 3-indole acetic acid to obtain rooted plantlets. The culture medium was solidified at a 45° angle, and the tubes were covered with cotton [11]. The cultures were maintained for 21 days in a culture chamber at 25 ± 2 °C under cool white light (LED lamps) with an irradiance of 26 μmol m^−2^ s^−1^ and a photoperiod of 16 h light/8 h dark.

*Baccharis conferta* shoots that were twenty-one days old (3 cm) were grown in Magenta vessels containing semisolid Murashige and Skoog (MS) culture medium with 30 g L^−1^ sucrose and 2.2 g L^−1^ phytagel^®^ without any plant growth regulators [14]. These cultures were maintained under the same conditions as previously described.

### 2.2. Co-Culture Experiments

Co-culture system: Twenty-one days old plants of *C. tenuiflora* and *B. conferta* were placed in Magenta vessels with semisolid MS medium, 30 g L^−1^ sucrose, and 2.2 g L^−1^ phytagel^®^ without any plant growth regulators. One plantlet of each species was positioned 1 cm apart from the other [15].

Three experimental groups were tested and are as follows: (1) *C. tenuiflora* co-cultured with *B. conferta* (Ct-Bc); (2) *C. tenuiflora* growing axenically without a host (Ct), with one plantlet placed in each Magenta vessel; and (3) *B. conferta* growing axenically without a parasite (Bc), also with one plantlet placed in each Magenta vessel. These cultures were maintained under the same conditions as described above. All analyses were conducted after 4 weeks.

Plant growth analysis: Morphological indices were evaluated using 11 plants from each group. The following variables were measured: plant height (cm), root length (cm), root-to-shoot ratio, and fresh weight (g) of both the aerial part and the root.

### 2.3. Environmental Scanning Electron Microscopy (ESEM)

Root sections of approximately 1 cm were cut and fixed in a solution (formaldehyde (10 mL), glacial acetic acid (5 mL), ethanol (50 mL), milli-Q water (35 mL), and sucrose (0.8 g)) for 24 h. Subsequently, three washes were performed with a phosphate buffer at pH 7.2 (0.2 M). Then, the dehydration process was conducted through successive immersions in ethanol at increasing concentrations (30%, 50%, 70%, 80%, and 90%). Between each ethanol immersion, the samples were allowed to stand for one hour. Finally, they were stored in 90% ethanol. Samples were placed on aluminum stubs with double-sided carbon adhesive tape and observed under an Environmental Scanning Electron Microscope (EVO LS10, Zeiss, Oberkochen, Germany). All observations were conducted under constant operating conditions: a beam acceleration voltage of 20 kV, a backscattered electron detector, and a water vapor pressure of 80 Pa. Images were captured at 2048 × 1536 pixels and stored in TIFF format. At least 10 root sections for each treatment were analyzed.

### 2.4. Confocal Laser Scanning Microscopy (CLSM)

**Figure 1:**
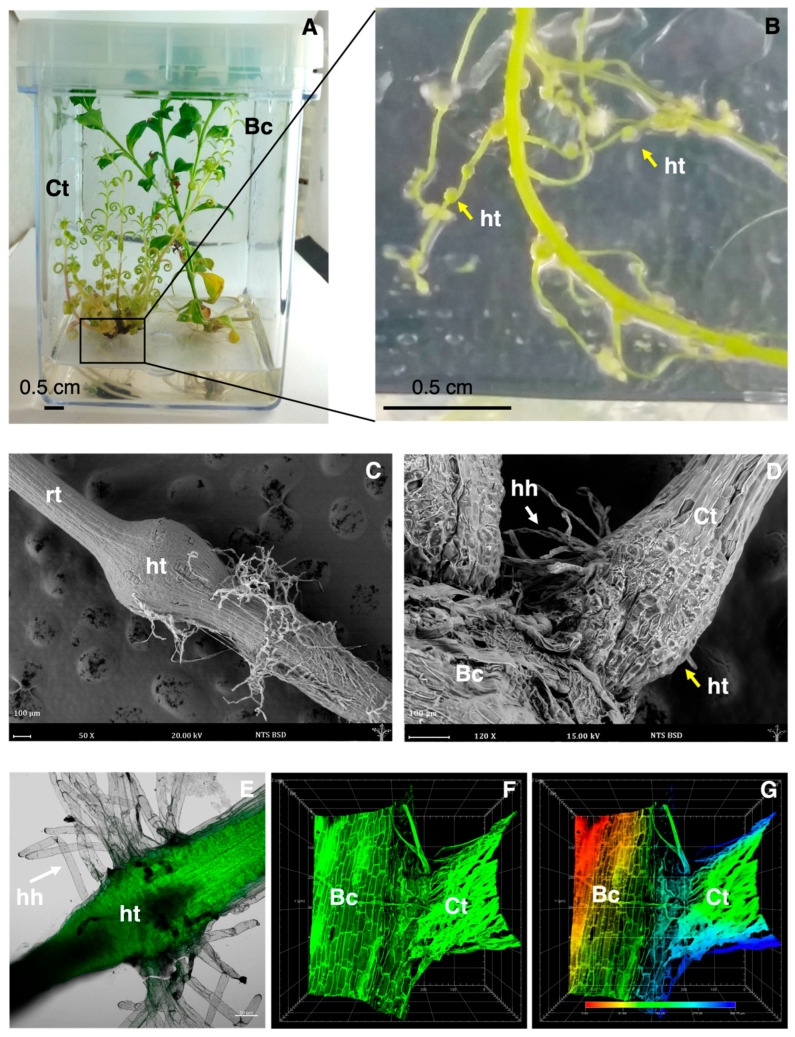
Representative images of the in vitro co-culture of *Castilleja tenuiflora* and *Baccharis conferta* (**A**) and plants of *C. tenuiflora* grown in co-culture showing the development of haustoria in the roots (**B**). Environmental Scanning Electron Microscopy micrographs of the roots of *C. tenuiflora* showing the development of haustorium (**C**) and the attachment to the roots of *B. conferta* (**D**). Laser scanning confocal microscopy micrographs of the haustoria of *C. tenuiflora* (**E**) developed in co-culture with *B. conferta*; (**F**,**G**) roots of *C. tenuiflora* and *B. conferta* attached in co-culture by haustorial bridge. Bc: *Baccharis conferta*; Ct: *C. tenuiflora*; ht: haustorium; hh: haustorial hairs; rt: roots. The yellow arrows indicate haustoria (ht), and the white arrows indicate haustorial hairs (hh).

Samples were observed using a Confocal Laser Scanning Microscope (LSM 800, Zeiss, Oberkochen, Germany). Root tips were fixed as described previously, and then the samples were mounted on glass slides and examined in lambda mode (a sequence of images collected at different wavelengths) at laser wavelengths of 405 nm and 488 nm with a 5% capacity. Figure 1F,G were captured in Z-stack mode, resulting in 36 slices with a 1.7 µm separation. Figure 1E was created using two channels (confocal and ESID). The images were produced using the ZEN 2.3 Blue edition software, and all micrographs were taken using Carl Zeiss’s EC Plan-Neofluar 20×/0.50 M27 objective.

### 2.5. Metabolite Analysis Through HPLC and LC-MS

Plant material (aerial parts and roots) was collected from each treatment, oven-dried at 40 °C for 48 h, and ground using a mortar and pestle. Subsequently, a microextraction of the whole plant material (1 g of dry matter (DM) in 50 milliliters of methanol) was conducted through sonication in methanol for 30 min and vacuum-filtered using Millipore^®^ membranes (0.20 μm). The filtrate was concentrated under reduced pressure with a rotary evaporator (V-250, Buchi, Flawil, Switzerland) operating at 210 mbar at 40 °C and 50 rpm. Finally, the samples were frozen at −20 °C for 24 h and freeze-dried for 3 h. For the HPLC and LC-MS analyses, the extracts were dissolved in HPLC-grade methanol (MeOH) at a concentration of 6 mg mL^−1^, sonicated for 30 min, filtered through a nylon acro disk (0.45 μm), and placed in a transparent glass vial.

LC-MS analysis was conducted using a Shimadzu LCMS 2020 system (Shimadzu, Tokyo, Japan) equipped with an electrospray ionization (ESI) source and LC-MS Labsolutions software, version 5.0.

For the analysis of *B. conferta*, we used an RP-18 column (Lichrospher 100. 250 × 4 mm, RP 18, 5 μm) (Merck, Darmstadt, Germany) connected to a column guard at 36 °C. The mobile phase consisted of 0.5% acetic acid aqueous solution as solvent A and acetonitrile as solvent B at a flow rate of 0.9 mL min^−1^. Gradient elution was conducted as follows: 0–1 min, 100–0% A–B; 1–3 min, 95–5% A–B; 3–20, 70–30%, A–B; 20–23 min, 50–50% A–B; 23–25 min, 20–80% A–B; 25–27 min, 0–100%·A–B; 27–30 min, 100–0% A–B [14].

*C. tenuiflora* was analyzed at 40 °C using a reverse-phase Chromolith^®^ High-Resolution RP-18 column (100 mm × 4 mm, 5 μm) (Merck, Darmstadt, Germany). The mobile phase consisted of Milli-Q water as solvent A and acetonitrile as solvent B. The gradient system was as follows: 0–2 min, 100–0% A–B; 2–5 min, 90–10% A–B; 5–10, 85–15%, A–B; 10–14 min, 80–20% A–B; 14–18 min, 75–25% A–B; 18–23 min, 70–30%·A–B; 23–27 min, 0–100% A–B; 27–30 min, 100–0% A–B. The injection volume was set to 20 μL, and the flow rate was 1 mL min^−1^.

Detection occurred within the 200–400 nm wavelength range. Mass spectra were recorded in the negative ion mode under the following operating conditions: 50–1000 m/z scan; N_2_ drying gas at 10 L min^−1^; nebulizer gas flow at 1.54 L min^−1^; interface voltage at 4.5 kV; and detector voltage at 1.2 kV.

### 2.6. Analysis of Differential Abundance of Metabolites

The metabolites analyzed in this study were selected based on their pharmacological significance. The fingerprints analyzed for *B. conferta* included phenolics (3-*O*-caffeoylquinic acid, caffeic acid, and 4,5-di-*O*-caffeoylquinic acid) and flavonoids (hispidulin, cirsimaritin, acacetin, pectolinarigenin, and 6-methoxykaempferide) [13]. In contrast, for *C. tenuiflora*, they comprised iridoids (aucubin and bartsioside), phenylethanoid glycosides (verbascoside and isoverbascoside), and lignans (tenuifloroside, sesamin, eudesmin, magnolin, and kobusin) [7]. Compounds were identified based on the determination of their retention time, ion *m*/*z* [M+H]-, and UV–Vis spectra, according to the literature reports, and according to compounds previously detected or isolated from *B. conferta* or *C. tenuiflora* (Tables S1 and S2). The identification was additionally confirmed for most compounds using available reference chemical standards.

The relative abundance of compounds was estimated based on the area of each peak and its contribution to the total signal area, as indicated by the chromatogram. The chromatogram’s raw data files were processed manually. The peak picking of each metabolite was performed with a 0.2 min retention time tolerance. The absorbance measurement was performed at 205 nm for iridoids [7], 280 nm for flavonoids and lignans [7,13], 325 nm for caffeic acid and caffeoylquinic acids [17], and 330 nm for PhGs [7]. Normalized peak intensity was logarithmically (Log_10_) transformed for further analysis. Differences in metabolite levels between axenic and co-culture were identified using a *t*-test and *p*-value as univariate statistical analysis. Metabolites with a *p*-value < 0.05 and a fold change (FC) ≥ 1.5 or FC ≤ 0.5 were considered differentially accumulating metabolites (DAMs) [18].

### 2.7. Statistical Analysis

Data is presented as the mean ± standard error of the mean (SEM). The normal distribution was confirmed using the Shapiro–Wilk test. Unpaired Student’s *t*-test and *p*-value were used to examine significant differences between two conditions (axenic vs. co-culture). *p*-values less than 0.05 (*p* < 0.05) were considered statistically significant. All statistical analyses were performed using GraphPad Prism version 10.4.2 for macOS (GraphPad Software, Boston, MA, USA).

## 3. Results

### 3.1. Formation of Haustorium in Co-Culture

An in vitro co-culture system was used to investigate the effects of host–parasite interaction between *B. conferta* and *C. tenuiflora* (Figure 1A). *C. tenuiflora*, when co-cultured with *B. conferta*, formed stronger roots (Figure 1B) with lateral protuberances (Figure 1B), which, based on previous experience, should correspond to haustoria [10,11].

The analysis conducted using ESEM (Figure 1C,D) and confocal microscopy (Figure 1E–G) confirmed the induction of *C. tenuiflora* haustoria under co-cultivation conditions. According to the ESEM analysis, haustoria measured between 500 and 900 µm in diameter (Figure 1C), and haustorial hairs were also formed (Figure 1D), which confirmed the plant’s attachment.

Figure 1E shows the roots from *C. tenuiflora* plants in co-cultivation conditions and the formation of globular haustoria with haustorial hairs using confocal microscopy, and the establishment of vascular connections under co-cultivation conditions (Figure 1F). In Figure 1G, using the “Depth Coding” tool (red, green, and blue), it was demonstrated the physical contact between the roots of *C. tenuiflora* and *B. conferta* by differentiating them with distinct color tones: reddish hues indicating proximity to the base of the stage (0 μm), and bluish hues corresponding to a higher position of the first root. This confirmed the presence of a zone where both roots interact in the same plane (indicated by green).

### 3.2. Growth in Co-Culture

**Figure 2:**
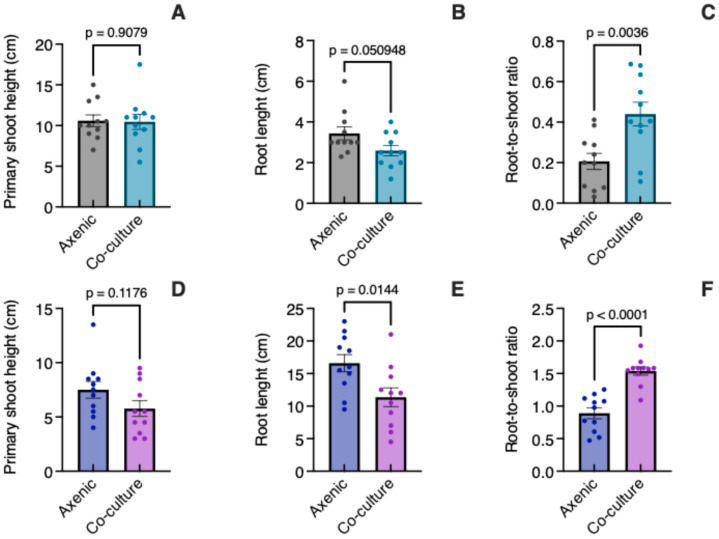
*Castilleja tenuiflora* (**A**–**C**) and *Baccharis conferta* (**D**–**F**) growth in axenic culture and co-culture. The root-to-shoot ratio is the ratio of root fresh weight to shoot fresh weight. Data are expressed as mean ± standard error (*n* = 11). Significant differences between axenic and co-culture plants were determined using an unpaired Student’s *t*-test (*p* < 0.05).

The height of *C. tenuiflora* plants was similar (*t* = 0.1171, *p* = 0.9079) in both axenic culture and co-culture with *B. conferta* (Figure 2A). Although the roots were a little shorter (*t* = 2.077, *p* = 0.0509) in the co-culture (Figure 2B), their biomass was higher (53%), which aligns with the significant difference (*t* = 3.296, *p* = 0.0036) in the root-to-shoot ratio observed in a co-culture compared to that for axenic conditions (Figure 2C). However, the total fresh biomass of *C. tenuiflora* only increased by 18.7% in a co-culture relative to axenic plants.

**Figure 3:**
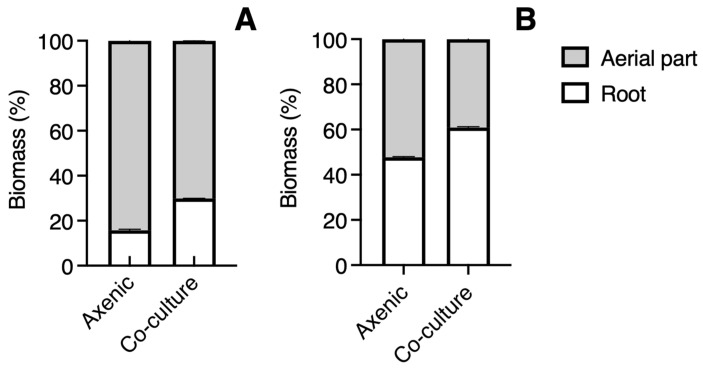
Proportion of total fresh biomass from aerial part and roots of *Castilleja tenuiflora* (**A**) and *Baccharis conferta* (**B**) in axenic culture and co-culture conditions.

The plants of *B. conferta* were similar in height in a co-culture to those grown axenically (*t* = 1.636, *p* = 0.1176) (Figure 2D). In co-culture, their roots were significantly shorter (*t* = 2.680, *p* = 0.0144) (Figure 2E) and still exhibited greater biomass (26%), highlighting a significant difference in the root-to-shoot ratio (*t* = 6.202, *p* < 0.0001) (Figure 2F). The proportion of root biomass in *C. tenuiflora* (14%) was half in axenic conditions than in co-culture conditions (30%) (Figure 3A). For *B. conferta*, the proportion of root biomass was also higher when in a co-culture with its host, reaching almost 60% (Figure 3B). No symptoms of adverse effects such as wilting, chlorosis, or necrosis were observed in *B. conferta* plants grown under co-culture.

### 3.3. Specialized Metabolites in Co-Culture

The LC-MS analysis was performed to relatively quantify specialized metabolites in *C. tenuiflora* and *B. conferta* grown under axenic and co-culture conditions. This analysis resulted in semi-quantitative profiling of 17 selected chemical compounds. The aim was to evaluate the impact of plant–plant interaction on the specialized metabolism of *C. tenuiflora* and its host, *B. conferta*.

PhGs were the most abundant metabolites in *C. tenuiflora*, followed by iridoids and lignans (Table S3). Two phenylethanoid glycosides (PhGs)—isoverbascoside and verbascoside—were identified (peaks 3 and 4, Figure S3). The two iridoids included aucubin (peak 1, Figure S1) and bartsioside (peak 2, Figure S1), where aucubin was the most abundant. For lignans, magnolin (peak 6, Figure S2) and tenuifloroside (peak 5, Figure S1) predominated over eudesmin (peak 7, Figure S2), kobusin (peak 8, Figure S1), and sesamin (peak 9, Figure S1).

In *B. conferta*, caffeoylquinic acids predominated over caffeic acid (Table S4); 4,5-di-*O*-caffeoylquinic acid (peak 3, Figure S7) constituted the most abundant metabolite, followed by chlorogenic acid (peak 1, Figure S7). For flavonoids, cirsimaritin (peak 5, Figure S7) and 6-methoxykaempferide (peak 8, Figure S7) predominated over hispidulin (peak 4, Figure S7), acacetin (peak 6, Figure S7), or pectolinaringenin (peak 7, Figure S7). In both species, the overall profiles were consistent across the two culture conditions (axenic and co-culture), with variations observed only in their relative abundance levels.

**Figure 4:**
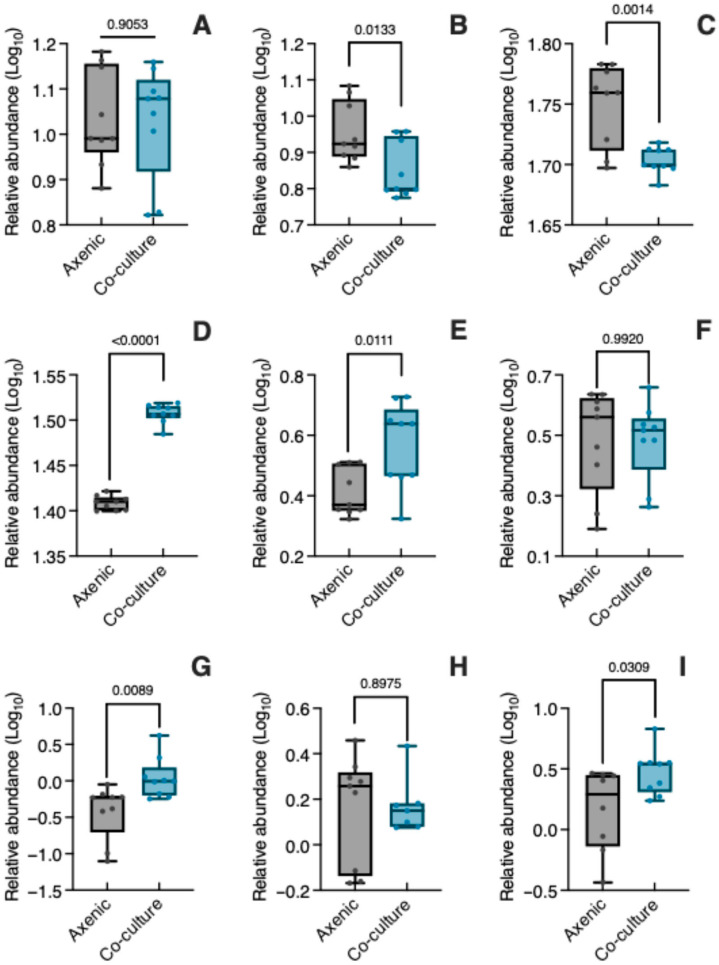
Relative abundance of specialized metabolites in *Castilleja tenuiflora* when growing axenically and in co-culture. (**A**) aucubin; (**B**) bartsioside; (**C**) isoverbascoside; (**D**) verbascoside; (**E**) tenuifloroside; (**F**) magnolin; (**G**) edudesmin; (**H**) kosubin; (**I**) sesamin. Relative abundance was log_10_ transformed. Significant differences between axenic and co-culture plants were determined using an unpaired Student’s *t*-test (*p* < 0.05).

Figure 4 presents the relative abundance (log_10_ transformed) of the nine metabolites produced by *C. tenuiflora* when grown axenically and in co-culture with its host. As mentioned before, aucubin is the most abundant iridoid in *C. tenuiflora*, and its relative abundance was unaffected by co-culture conditions (*t* = 0.1209, *p* = 0.9053) (Figure 4A, Table S3). In contrast, bartsioside (Figure 4B, Table S3) decreased significantly in co-culture (*t* = 2.782, *p* = 0.0133) compared to axenic culture. Both PhGs changed significantly, with isoverbascoside decreasing (*t* = 3.866, *p* = 0.0014) while verbascoside increased (*t* = 22.39, *p* < 0.0001) (Figure 4C,D, Table S3).

Like iridoids and PhGs, lignans changed differentially in axenic and co-culture conditions (Figure 4E–I, Table S3). Tenuifloroside (*t* = 2.871, *p* = 0.0111), eudesmin (*t* = 2.974, *p* = 0.0089) and sesamin (*t* = 2.382, *p* = 0.0309) increased significantly under co-culture, with magnolin (*t* = 0.0101, *p* = 0.9920) and kobusin (*t* = 0.1311, *p* = 0.8975) having similar relative abundances in both culture conditions.

Among DAMs, the analysis indicated that the lignans eudesmin (2.38-fold change) and sesamin (1.78-fold change) were expressed differentially in *C. tenuiflora* when parasitizing *B. conferta*.

**Figure 5:**
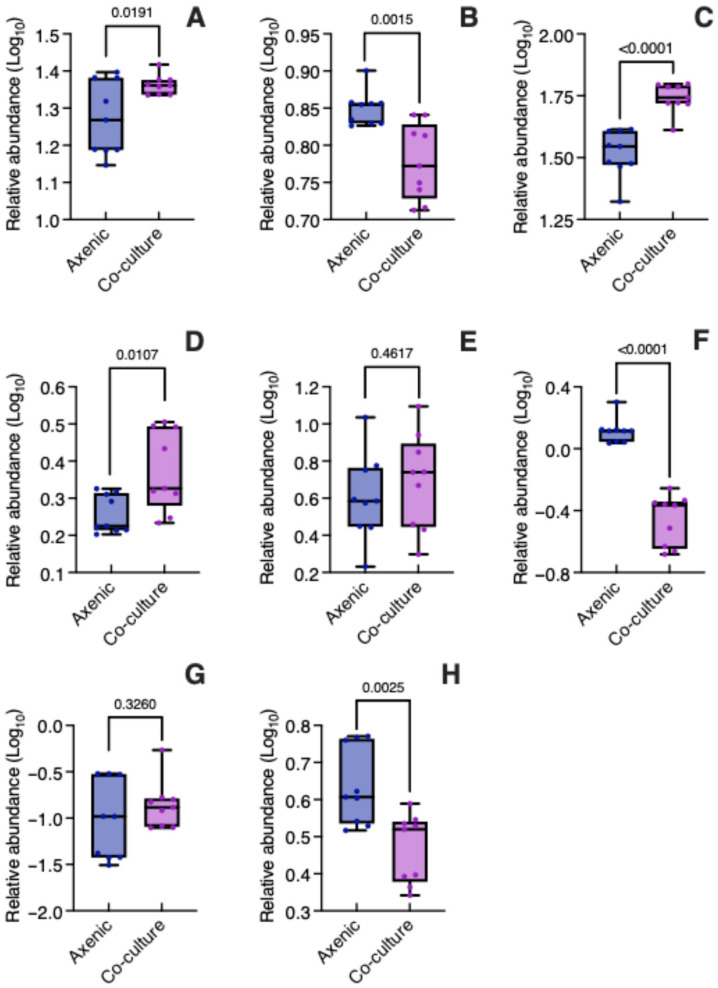
Relative abundance of specialized metabolites in *Baccharis conferta* when grown axenically and in co-culture. (**A**) chlorogenic acid; (**B**) caffeic acid; (**C**) 4,5 di-*O*-caffeoylquinic acid; (**D**) hispidulin; (**E**) cirsimaritin; (**F**) acacetin; (**G**) pectolinarigenin; (**H**) 6-methoykaempferide. Relative abundance was log_10_ transformed. Significant differences between axenic and co-culture plants were determined using an unpaired Student’s *t*-test (*p* < 0.05).

The relative abundance (log_10_ transformed) of the eight metabolites found in *B. conferta* is presented in Figure 5. The two caffeoylquinic acids, namely chlorogenic acid (*t* = 2.606, *p* = 0.0191) (Figure 5A, Table S4) and 4,5-di-*O*-caffeoylquinic acid (*t* = 6.005, *p* < 0.0001) (Figure 5C, Table S4), were significantly more abundant in *B. conferta* when co-cultured with *C. tenuiflora* compared to growing axenically. In contrast, caffeic acid was more abundant in the axenic condition (*t* = 3.822, *p* = 0.0015) (Figure 5B, Table S4). Given that caffeic acid is a precursor of phenylethanoid glycosides, its potential translocation to *C. tenuiflora* was investigated by assessing its presence in both the root and aerial parts, using LC-MS. However, it was not possible to determine its relative abundance due to coelution with phenylethanoid glycosides (found at a retention time of 7.01 min, Figure S5).

The relative abundance of hispidulin (*t* = 2.888, *p* = 0.0107) (Figure 5D, Table S4) increased significantly in co-culture, while acacetin (*t* = 9.517, *p* < 0.0001) (Figure 5F, Table S4) and 6-methoxykaempferide (*t* = 3.588, *p* = 0.0025) (Figure 5H, Table S4) significantly decreased. The relative abundance of cirsimaritin (*t* = 0.7542, *p* = 0.4617) (Figure 5E, Table S4) and pectolinaringenin (*t* = 1.013, *p* = 0.3260) (Figure 5G, Table S4) was similar in both axenic and co-culture conditions.

Among DAMs, the analysis indicated that 4,5-di-*O*-caffeoylquinic acid (1.56-fold change) and acacetin (0.26-fold change) were expressed differentially in *B. conferta* when parasitized by *C. tenuiflora*.

## 4. Discussion

Our results indicate that *C. tenuiflora* develops lateral haustoria when co-cultured in vitro with *B. conferta.* The haustoria resemble those observed in wild plants [10] and those produced in vitro after applying HIFs such as H_2_O_2_ or vanillin [11]. The formation of haustorial hairs was also observed, which is essential for efficient parasitism [19].

The formation of haustoria involves four distinct phases: recognition, attachment, invasion, and connection [2]. In the rhizosphere, host plants release molecules that work as host recognition signals. Among these molecules, strigolactones (SLs) have received special attention as signaling compounds for plant–plant interaction [20,21]. So far, the composition of the root exudates of *B. conferta* or other species of this genus has not been elucidated. The characterization of the root exudates of several species in the family Asteraceae (which includes *Baccharis*) revealed the presence of SLs, with orobanchyl acetate and 5-deoxystrigol as the significant components [22]. Recently, using the SL analog GR24 5DO, it has been proposed that *Castilleja foliolosa* possesses a large clade of KAI2d proteins, which are receptors for exogenous SLs and are related to the formation of haustorium-like structures [23], as noted for other facultative hemiparasites. It is known that the parasite recognizes the host with high specificity, and that these proteins exhibit high sensitivity and specificity in recognizing specific mixtures of SLs, which enables them to discriminate between host and non-host plants, resulting in the formation of haustoria [24]. Therefore, it can be hypothesized that strigolactones have a signaling role in the establishment of the host–parasite interaction in *B. conferta*-*C. tenuiflora* co-culture.

To establish the co-culture system, it was essential to promote root development in both species, which, based on previous experience, was achieved using SH medium for *C. tenuiflora* and MS medium for *B. conferta.* For the co-culture experiments, MS medium was selected, as it provides a higher nitrogen content compared to SH medium (60 mM vs. 27.4 mM) [25], thus helping to prevent a possible negative effect of *C. tenuiflora* on *B. conferta* survival. Previous studies have shown that parasitism tends to increase under nitrogen-deficient conditions; however, such conditions may also compromise host viability [26]. Environmental factors—including water availability, light, and nutrient supply, particularly nitrogen and phosphorus, along with their interactions—have been proposed to influence the dynamics between host and parasite [27,28,29]. Consequently, the performance of both species is expected to differ under culture conditions other than MS. The specific effects of nutrient supply on this interaction remain an open question and should be addressed in future studies.

Plant height of *B. conferta* under co-culture and axenic conditions was statistically similar, and no symptoms of adverse effects were observed. In contrast, root biomass yield was higher for both the host and the parasite in a co-culture, corresponding to a significant difference in the root-to-shoot ratio. As observed in the root hemiparasite *Rhinanthus alectorolophus* (Orobanchaceae), environmental conditions affect both the parasitic and autotrophic components, and the hemiparasite’s photosynthesis plays a role in the assimilation of resources from the host [30].

Host type, host’s growth rate, and resistance to the parasite all influence the parasite’s growth [31,32]. A field study observed that the physiology of *C. applegatei* was affected by the host type; however, this did not impact the community due to the presence of N-fixing hosts [32]. Furthermore, apparent competition for resources was observed in the co-culture, which may explain why the total biomass of both species was similar. In a parasitic–host system, the mass is typically lower due to its reduction by the parasite compared to that by the host when grown alone [31].

In the parasitic relationship between *Struthanthus flexicalulis* (Loranthaceae) and *Baccharis dracunculifolia*, parasitism caused significant physiological imbalances that adversely affected the host’s development [33]. The resistance of potential hosts to infection does not necessarily prevent the hemiparasite from forming haustoria and establishing a connection with its host. This resistance may be reflected as a form of relative tolerance, allowing the host to experience minimal impact on its growth and development, while simultaneously enabling the hemiparasite to develop properly [34]. In this context, variations in the parasitized *B. conferta* are not significant, suggesting that it has developed a certain degree of resistance that allows it to tolerate infection by *C. tenuiflora*, which may be associated with potential non-nitrogen limitation conditions (as provided by the MS culture medium), already discussed. This result is consistent with observations made in *B. conferta* natural populations, where no significant variations in height and chlorophyll content between parasitized and non-parasitized plants were found [10]. In this regard, it has been proposed that, through co-evolution, native hosts have developed tolerance to native parasites, as seen in the cases of *B. conferta* and *C. tenuiflora*. It is hypothesized that the mechanisms of tolerance include a poor haustorial connection [35].

*Castilleja tenuiflora*, growing on *B. conferta*, enhances its accumulation of PhGs and lignans, which share a common origin in the shikimate pathway, while reducing the accumulation of iridoids synthesized via the terpenes pathway. Among the molecules identified as signals of plant–plant interactions, H_2_O_2_ is recognized as crucial for haustorial induction and, consequently, the establishment of parasitic interactions [36]. Our research group has previously reported that haustorium formation in *C. tenuiflora* correlates with the accumulation of H_2_O_2_ [11]. Additionally, we observed that eliciting *C. tenuiflora* plantlets with H_2_O_2_ increased the total content of phenolic compounds, particularly promoting the synthesis of lignans. We discovered that H_2_O_2_ upregulates key enzyme-encoding genes (PAL, TyrDC, GOT2, AO3, ADD, and CHS) involved in the phenolic pathway [37]. The connection between signaling molecules of plant–plant interactions and specialized metabolism represents an interesting research topic for further exploration.

The in vitro co-culture of *B. conferta* with its host or parasite differentially influenced the synthesis of specialized metabolites. Specifically, the relative abundance of the caffeoylquinic acids, chlorogenic acid and 4,5-diQ, increased while that of caffeic acid decreased. Similarly, the relative abundance of the flavonoid acacetin diminished. The decrease in caffeic acid concentration is of particular interest, given its role as a biosynthetic precursor of the PhGs found in *C. tenuiflora*. Although its potential translocation was investigated, quantification was not feasible due to coelution with verbascoside, which was markedly more abundant. Moreover, the experimental design did not account for the possibility of interspecific metabolite transfer, suggesting the need for further investigation.

The translocation of specialized metabolites between interacting plants has been documented, such as the case of flavonoid transfer from *Mangifera indica* to its hemiparasite *Dendropthoe falcata* [38], the translocation of isoquinoline alkaloids to the hemiparasite *Tristerix verticillatus* from its host *Berberis montana* [39], or the uptake of quinolizidine alkaloids by *C. miniata* and *C. indivisa* from *Lupinus* [40]. Parasitism of *S. flexicaulis* on *B. dracunculifolia* affected flavonoid balance, as the parasitized individuals exhibited lower phenolic content than their non-parasitized counterparts [33]. Similarly, the production of flavonoids in the leaves of *Theobroma cacao* L. decreased due to the hemiparasitic species *Acanthosyris paulo-alvinii* [41]. In the hemiparasite *Psittacanthus schiedeanus* (Loranthaceae), RNA-seq methodology identified differentially expressed genes related to synthesis, signaling, homeostasis, and response to auxin and jasmonic acid synthesis, showing the complexity of the molecular events involved in the host–parasite relationship [42]. Besides other roles, jasmonates have been implicated in signaling the biosynthesis of flavonoids [43].

Overall, co-culture conditions predominantly enhanced differentially the production of phenolic-type compounds in both species: caffeoylquinic acids in *B. conferta* and lignans in *C. tenuiflora.* Chlorogenic acids (CGAs), a subclass of caffeoylquinic acids, play a role in chemical defense mechanisms due to their strong antioxidant capacity. They also serve as intermediates in the lignin biosynthetic pathway, as they can be remobilized for monolignol synthesis and contribute to restricting pathogen invasion [44,45]. Considering that parasitism imposes stress on the host plant, the observed increase in CGAs in *B. conferta* may reflect a defensive response to parasitization, which is consistent with its resistance to *C. tenuiflora*.

In *S. hermonthica*, an obligate parasitic plant, it has been reported that exogenous quinones, acting as HIFs, transcriptionally activate genes involved in monolignol biosynthesis. Moreover, external monolignols, such as coniferyl alcohol, are incorporated into the pre-haustorial cell wall. These findings supported the hypothesis that monolignols play a dual role, acting both as signaling molecules for haustorium induction and as structural precursors for lignin deposition during haustorium development [46]. Coniferyl alcohol, a key monolignol derived from the phenylpropanoid pathway, is a common precursor for both lignin and lignan biosynthesis [47]. Therefore, the observed increase in lignans, such as eudesmin and sesamin, in *C. tenuiflora* grown in co-culture with its host may reflect an upregulation of monolignol biosynthesis associated with the parasitic process.

The co-culture system offers distinct advantages over field-based approaches, particularly given the inherent fragility of parasitic roots and the reversible nature of haustoria formation. This in vitro model provides a controlled environment that enables detailed investigation of the complex physiological and molecular interactions between hosts and parasites. Elucidating the regulatory mechanisms governing haustorium formation may significantly advance our understanding of plant parasitism and its underlying biology. Moreover, this system could facilitate biotechnological efforts aimed at domestication and cultivation of *C. tenuiflora*, while enabling the optimization of physiological parameters required to maximize its production of therapeutically relevant specialized metabolites.

## 5. Conclusions

Induction of haustoria in *C. tenuiflora* is feasible under an in vitro co-culture with *B. conferta*. The development and chemical profiles of *B. conferta* and *C. tenuiflora* change during the co-culture due to the host–parasite interaction. Research currently being performed in our laboratory explores the genetic basis of haustorium formation, among other questions.

## Data Availability

The experimental data used to support the results are included in the article and Supplementary Material; further inquiries can be directed to the corresponding author.

## Supplementary Materials

The following supporting information can be downloaded at https://www.mdpi.com/article/10.3390/biology14080990/s1, Table S1: Chemical compounds identified in *C. tenuiflora* by LC-PDA-ESI-MS; Table S2: Chemical compounds identified in *Baccharis conferta* by LC-PDA-ESI-MS; Table S3: Comparative relative area under the curve (AUC) values of detected compounds in *C. tenuiflora* grown under axenic and co-culture; Table S4: Comparative relative area under the curve (AUC) values of detected compounds in *B. conferta* grown under axenic and co-culture; Figure S1: Representative chromatogram of *C. tenuiflora* at 205 nm; Figure S2: Representative chromatogram of *C. tenuiflora* at 280 nm; Figure S3: Representative chromatogram of *C. tenuiflora* at 330 nm; Figure S4: Representative MS Spectra of the compounds identified in *C. tenuiflora*; Figure S5: HPLC chromatogram (A) of *C. tenuiflora* root extract at 325nm, UV spectra (B) and mass spectra (C) of peak at Rt = 7.01 min; Figure S6: Representative chromatogram of *B. conferta* at 280 nm; Figure S7: Representative chromatogram of *B. conferta* at 325 nm; Figure S8: Representative MS Spectra of the compounds identified in *B. conferta*.

## Abbreviations

The following abbreviations are used in this manuscript:

4,5diQ	4,5-di-*O*-caffeoylquinic acid
HIFs	haustorium-inducing factors
PhGs	phenylethanoid glycosides
