# Supplementary material for: Haustorium Formation and Specialized Metabolites Biosynthesis Using Co-Culture of Castilleja tenuiflora Benth. and Baccharis conferta Kunth
Source: Biology (Basel). 2025 Aug 4;14(8):990. doi: 10.3390/biology14080990 (PMC12383656; doi:10.3390/biology14080990)
Supplement: Supplementary file 1 [file biology-14-00990-s001.zip › biology-3750793-supplementary.pdf]

biology

MDPI

Article

Haustorium Formation and Specialized Metabolites Biosynthesis Using Co-culture of *Castilleja tenuiflora* Benth. and *Baccharis conferta* Kunth

Annel Lizeth Leyva-Peralta[1], José Luis Trejo-Espino[1,2], Guadalupe Salcedo-Morales[1], Daniel Tapia-Maruri[1,2], Virginia Medina-Pérez[1], Alma Rosa López-Laredo[1] and Gabriela Trejo-Tapia[1,2]*

[1]Instituto Politécnico Nacional, Centro de Desarrollo de Productos Bióticos, Departamento de Biotecnología, Yautepec, Morelos, 62739, México; annie.leyva.peralta@gmail.com (A.L.L.P); jtrejo@ipn.mx (J.L.T.E); gsalcedo@ipn.mx (G.S.M.); dmaruri@ipn.mx (D.T.M.); vmedinap@ipn.mx (V.M.P); arlopez@ipn.mx (A.R.L.L)
[2]Instituto Politécnico Nacional, Red de Biotecnología (G.T.T.), Red de Medio Ambiente (J.L.T.E., D.T.M., G.T.T), México
* Correspondence: gttapia@ipn.mx (G.T.T.)

Figure S1. Representative chromatogram of *C. tenuiflora* at 205 nm.

Figure S2. Representative chromatogram of *C. tenuiflora* at 280 nm.

Figure S3. Representative chromatogram of *C. tenuiflora* at 330 nm.

Figure S4 Representative MS Spectra of the compounds identified in *C. tenuiflora*

Figure S5. HPLC chromatogram (A) of *C. tenuiflora* root extract at 325 nm, UV spectra (B) and mass spectra (C) of peak at Rt=7.01 min. caffeic acid *m/z* 179; verbascoside *m/z* 623

Figure S6. Representative chromatogram of *B. conferta* at 280 nm.

Figure S7. Representative chromatogram of *B. conferta* at 325 nm.

Figure S8. Representative MS Spectra of the compounds identified in *B. conferta*

Table S3. Comparative relative area under the curve (AUC) values of detected compounds in *C. tenuiflora* grown under axenic and co-culture.

Table S4. Comparative relative area under the curve (AUC) values of detected compounds in *B. conferta* grown under axenic and co-culture.

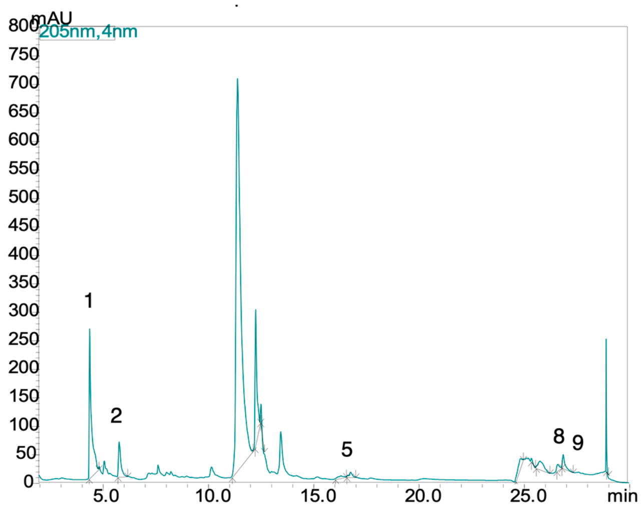

Figure S1. Representative chromatogram of *C. tenuiflora* at 205 nm. (1) aucubin, Rt=4.58 min; (2) bartsioside, Rt=5.97 min; (5) tenuifloroside, Rt=16.28 min; (8) kobusin, Rt=26.48 min; (9) sesamin, Rt=28.41 min.

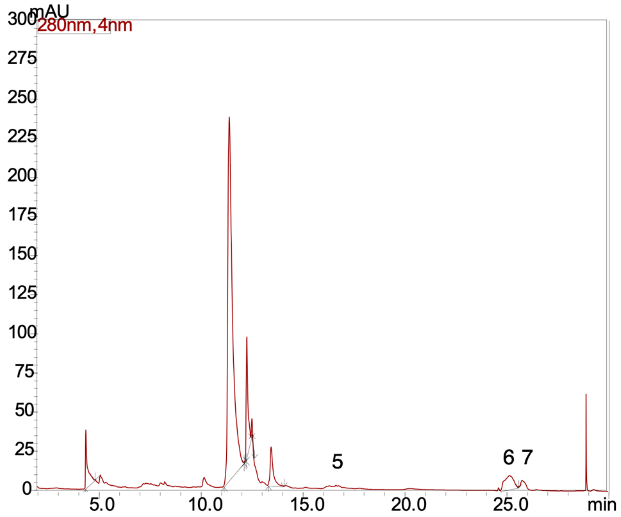

Figure S2. Representative chromatogram of *C. tenuiflora* at 280 nm. (5) tenuifloroside, Rt=16.28 min; (6) magnolin, Rt=25.17 min; (7) eudesmin, Rt=25.73 min.

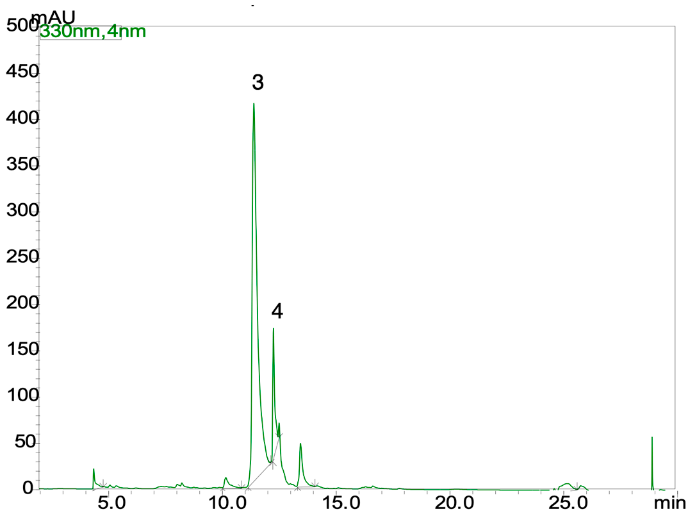

Figure S3. Representative chromatogram of *C. tenuiflora* at 330 nm. (3) isoverbascoside, Rt=11.26 min; (4) verbascoside, Rt=12.29 min

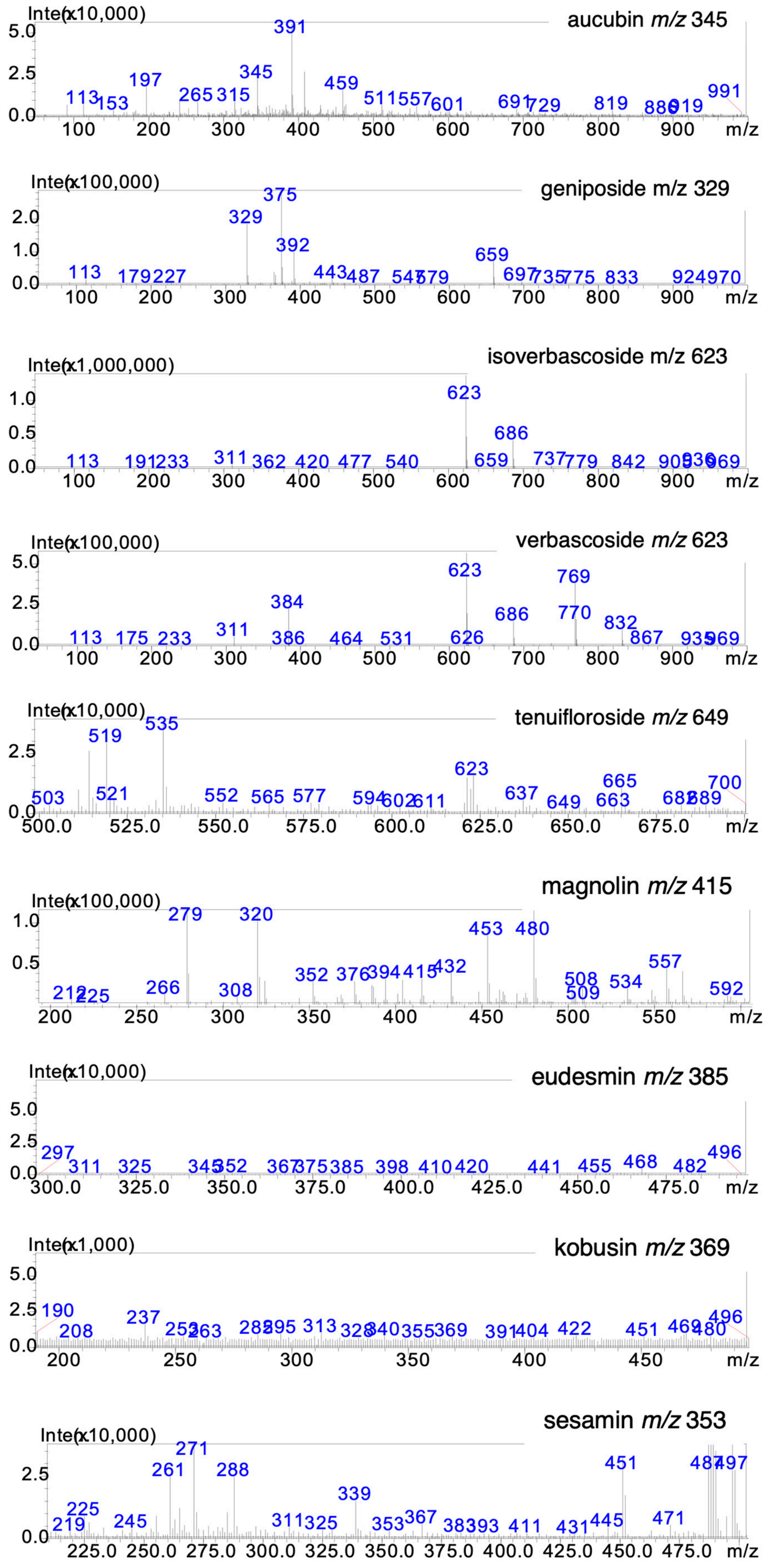

Figure S4. Representative MS Spectra of the compounds identified in *C. tenuiflora*

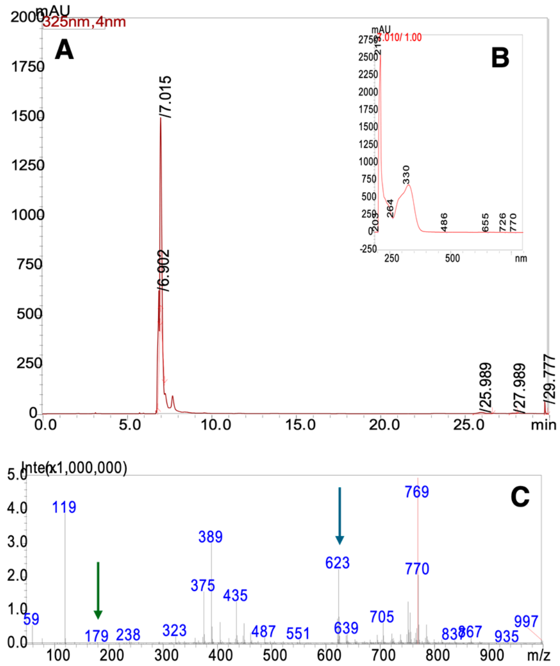

Figure S5. HPLC chromatogram (A) of *C. tenuiflora* root extract at 325 nm, UV spectra (B) and mass spectra (C) of peak at Rt=7.01 min. caffeic acid *m*/*z* 179; verbascoside *m*/*z* 623

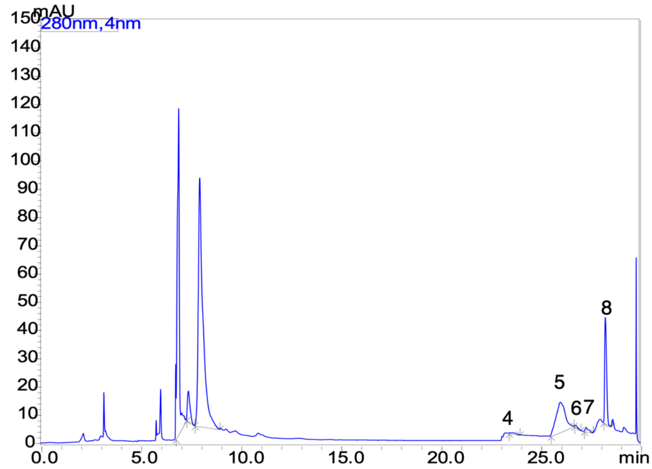

Figure S6. Representative chromatogram of *B. conferta* at 280 nm. (4) hispidulin, Rt = 23.35 min; (5) cirsimaritin, Rt = 25.9 min; (6) acacetin, Rt = 26.62 min; (7) pectolinaringenin, Rt = 27.12 min; (8) 6-methoxykaempferide, Rt = 27.98 min

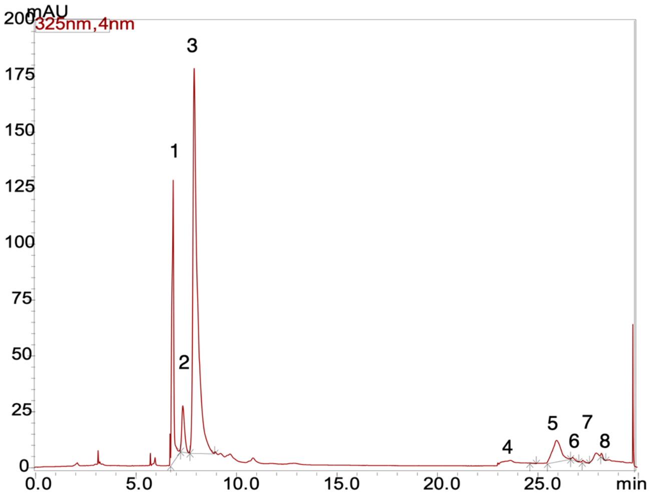

Figure S7. Representative chromatogram of *B. conferta* at 325 nm. (1) chlorogenic acid, Rt = 6.83 min; (2) caffeic acid, Rt = 7.13 min; (3) 4,5-di-O-caffeoylquinic acid, Rt = 27.98 min

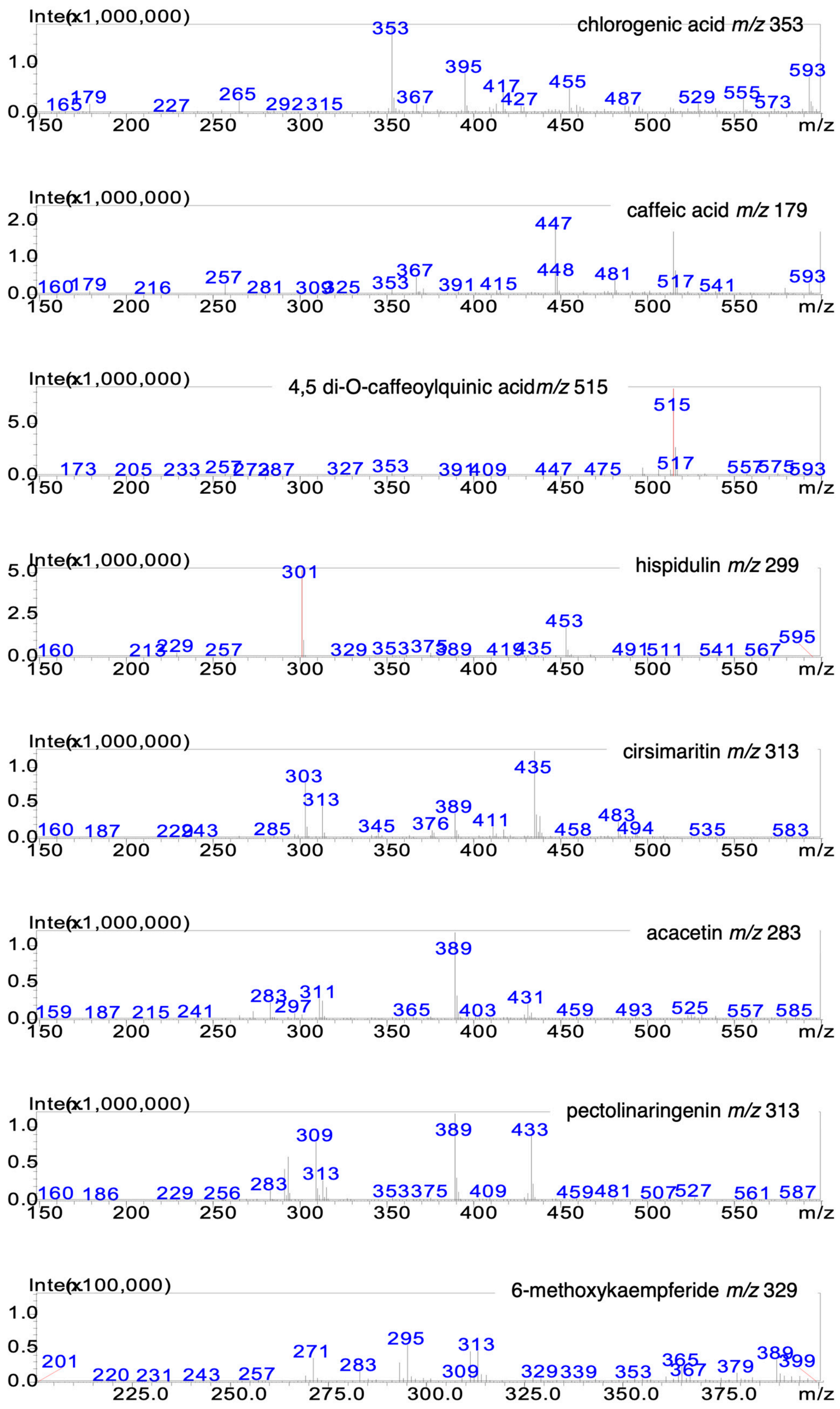

Figure S8. Representative MS Spectra of the compounds identified in *B. conferta*.

Table S3. Comparative relative area under the curve (AUC) values of detected compounds in *C. tenuiflora* grown under axenic and co-culture.

Compound	Class	AUC (mAU)	
		Axenic culture	Coculture
Aucubin	Iridoid	11.15±0.93	11.05±093
Bartsioside	Iridoid	9.15±0.61	7.17±0.44
Isoverbascoside	Phenylethanoid glycoside	56.31±1.43	50.54±0.42
Verbascoside	Phenylethanoid glycoside	25.62±0.15	32.15±0.26
Tenuifloroside	Lignan	2.62±0.16	3.85±0.38
Magnolin	Lignan	3.22±0.36	3.14±0.48
Eudesmin	Lignan	0.48±0.09	1.35±0.38
Kobusin	Lignan	1.62±0.25	1.54±0.20
Sesamin	Lignan	1.81±0.38	3.23±0.51

The quantities of the compounds are reported in milli-Absorbance Units, as not all the compounds were isolated to generate standard calibration curves for concentration determination. Data are expressed as mean ± ±standard error (n=9)

Table S4. Comparative relative area under the curve (AUC) values of detected compounds in *B. conferta* grown under axenic and co-culture.

Compound	Class	AUC (mAU)	
		Axenic culture	Coculture
Chlorogenic acid	Caffeoylquinic acid	19.19±1.46	23.07±0.47
Caffeic acid	Hydroxycinnamic acid	7.08±0.13	6.03±0.23
4,5-di-O-caffeoylquinic acid	Dicaffeoylquinic acid	33.66±2.24	55.65±2.37
Hispidulin	Flavonoid	1.82±0.07	2.43±0.20
Cirsimaritin	Flavonoid	4.58±0.90	5.71±1.11
Acacetin	Flavonoid	1.32±0.09	0.37±0.04
Pectolinaringenin	Flavonoid	0.14±0.04	0.17±0.04
6-methoxykaempferide	Flavonoid	4.43±0.36	2.99±0.21

The quantities of the compounds are reported in milli-Absorbance Units, as not all the compounds were isolated to generate standard calibration curves for concentration determination. Data are expressed as mean± ± standard error (n=9)
